# Identification and Characterization of a Gene *stp17* Located on the Linear Plasmid pBSSB1 as an Enhanced Gene of Growth and Motility in *Salmonella enterica* Serovar Typhi

**DOI:** 10.3389/fcimb.2016.00110

**Published:** 2016-10-05

**Authors:** Haifang Zhang, Yunxia Zhu, Xiaofang Xie, Min Wang, Hong Du, Shungao Xu, Ying Zhang, Mingyu Gong, Bin Ni, Huaxi Xu, Xinxiang Huang

**Affiliations:** ^1^Department of Clinical Laboratory, The Second Affiliated Hospital of Soochow UniversitySuzhou, China; ^2^School of Medicine, Jiangsu UniversityZhenjiang, China; ^3^Department of Clinical Laboratory, Shanghai East Hospital, School of Medicine, Tongji UniversityShanghai, China

**Keywords:** *Salmonella enterica* serovar Typhi, linear plasmid, pBSSB1, *stp17*, growth, motility, ATPase

## Abstract

The linear plasmid pBSSB1 mediates the flagellar phase variation in H:z66 positive *Salmonella enterica* serovar Typhi (*S.* Typhi). The gene named *stp17* (*S*. Typhi plasmid number 17 gene) is located on pBSSB1 and encodes the protein STP17. The expression pattern at the protein-level and function of STP17 remains unknown. In this study, the recombinant protein STP17_His6_ was expressed, purified and used to prepare the polyclonal anti-STP17 antibody. We detected protein-level expression of *stp17* in *S.* Typhi and further investigated the protein expression characteristics of *stp17* in different growth phases by western blot analysis. The effects of STP17 on bacterial growth and motility were analyzed. In addition, the structure of STP17 was predicted and the active site of STP17 was identified by site-directed mutagenesis. The results showed that STP17 was expressed stably in the wild type strain of *S*. Typhi. STP17 expression at the protein level peaks when cultures reach an OD_600_ value of 1.2. The growth rate and motility of the Δ*stp17* strain were significantly decreased compared with the wild type strain (*P* < 0.05) and this phenotype was restored in the *stp17* complementary strain. Moreover, the growth rate and motility of the *stp17* over-expression strain was greater than the wild type strain. STP17 contains nine Helix segments, six Stand segments and some Coil segments in the secondary structural level. The top-ranked 3-D structure of STP17 predicted by I-TASSER contains a putative ATPase domain and the amino acid residues of GLY16, GLY19, LYS20, ASN133, LYS157, and LYS158 may be the active site residues of STP17. Finally, STP17 was able to catalyze the ATP to ADP reaction, suggesting that STP17 may be an ATPase. To our knowledge, this is the first report describing the protein expression characteristics of STP17 in *S.* Typhi, showing that STP17 promotes bacterial growth and motility, which may be associated with its potential ATPase activity.

## Introduction

*Salmonella enterica* serovar Typhi (*S.* Typhi) is an important human pathogen responsible for typhoid fever (Everest et al., 2001; Wain et al., 2015). Harm caused by *S.* Typhi has been greatly reduced by development and use of antibiotics. However, typhoid fever remains a common disease in tropical and subtropical regions, with many drug-resistant strains having been isolated (John et al., 2016; Yap et al., 2016). In addition, *S.* Typhi is an important model organism for researching the expression and regulation of prokaryotic genes (Winter et al., 2014).

Most serovars of *S. enterica* contain *fliC* and *fljB* genes encoding the phase-1 and phase-2 flagellar antigen respectively at different chromosomal loci, and show biphasic characteristics (Simon et al., 1980). Biphasic serovars of *S. enterica* alternatively express two flagellar antigens through a process called “phase variation,” which is mediated by a *hin* located upstream *fljBA* (Henderson et al., 1999). *S.* Typhi normally do not possess flagellar antigen phase variation because they only harbor the *fliC* gene (H:d) and lack the *fljB* gene. However, some isolates of *S.* Typhi, from Indonesia contain an *fljB* gene equivalent (*fljB*^*z*66^) which encode the novel flagellin named H:z66 (Guinee et al., 1981). *fljB*^*z*66^ is located on linear plasmid pBSSB1 which is the first non-bacteriophage-related linear plasmid found in *Enterobacteriaceae* and mediates the unidirectional flagellar phase variation of the *S.* Typhi z66-positive strain (Baker et al., 2007a,b). *S*. Typhi z66-positive strain was isolated mainly from Indonesia and caused the incidences of serious typhoid fever which may be due to the escaping immunologic surveillance through its unique unidirectional flagellar phase variation (Baker et al., 2008; Hatta et al., 2011).

Thirty-three putative genes are encoded on pBSSB1 and most remain to be elucidated with the exception of three genes: 030, *fljB*^*z*66^, and *fljA*^*z*66^ (Baker et al., 2007a,b). The genes *fljB*^*z*66^ and *fljA*^*z*66^ have been well studied in terms of gene expression regulation and have been found to mediate the flagellar variation in z66-positive *S.* Typhi (Huang et al., 2004; Xu et al., 2008, 2010; Zou et al., 2009). The seventeenth gene, here named *stp17* (*S.* Typhi plasmid number 17 gene), is immediately adjacent to the possible replication origin of pBSSB1 and is predicted to encode a putative nucleotide binding protein STP17 (Baker et al., 2007a). In 2014, the expression characteristic of *stp17* in mRNA level was demonstrated (Zhao et al., 2014). However, the function of *stp17* has not previously been described. Here, we demonstrate that *stp17* may promote bacterial growth and motility through the ATPase activity of STP17.

## Materials and methods

### Bacterial strains, plasmids, and culture conditions

A z66-positive wild-type strain, *S*. Typhi GIFU10007, was used in this study. Mutants and plasmids used in this work are listed in Table 1. Primer sequences are described in Table 2. Bacteria were cultured in Luria–Bertani (LB) broth at 37°C.

**Table 1:** **Strains and plasmids used in the study**.

**Strain or plasmid**	**Relevant characteristics**	**Reference or source**
**STRAINS**		
*S*. Typhi GIFU10007	Wild-type strain, Z66^+^	Laboratory collection
SY372λpir	*E. coli* host strain of suicide plasmid	Laboratory collection
Δ*stp17*	GIFU10007(Δ*stp17*)	This work
Δ*stp17*(pACYC184)	Δ*stp17*containing pACYC184 empty vector	This work
Δ*stp17*(pACYC184*-stp17*)	Δ*stp17* containing pACYC184*-stp17*,recombinant plasmid	This work
wt(pBAD)	GIFU10007 containing pBAD, empty vector	This work
wt(pBAD-*stp17*)	GIFU10007 containing pBAD-*stp17*,recombinant plasmid	This work
DH5a(pET28b-*stp17*)	*E. coli* DH5 containing pET28b-*stp17*,recombinant plasmid	This work
BL21(pET28b-*stp17*)	*E. coli* BL21 containing pET28b*-stp17*,recombinant plasmid	This work
ΔpBSSB	GIFU10007 cured pBSSB1	This work
ΔpBSSB (pBAD)	ΔpBSSB containing pBAD empety vector	This work
ΔpBSSB (pBAD-*stp17*)	ΔpBSSB containing pBAD-*stp17* recombinant plasmid	This work
**PLASMIDS**		
pGMB151	Suicide plasmid; *sacB*; Amp^r^	Laboratory collection
pGMB-*stp17*	pGMB151 containing *stp17* deleted homologous fragments	This work
pACYC184	Complementary vector; Chlr, Tet^r^	Laboratory collection
pACYC184*-stp17*	pACYC184 containing *stp17* gene	This work
pBAD/gIII	Expression vector; Amp^r^	Laboratory collection
pBAD-*stp17*	pBAD/gIII containing *stp17* gene	This work
pET28b	Expression vector; Kana^r^	Laboratory collection
pET28b-*stp17*	pET28b containing *stp17* gene	This work

**Table 2:** **Primers used in this study**.

**Primers**	**Sequence(5′-)**	**Purpose**
F1A(*BamH* I)	TTAGGATCCAGTTCCGAATCCCATAGGC	*stp17* mutant construction
F1B	CGAATAGATAACACCTCCCTTATAGTTCCA	
F2A	AGGGAGGTGTTATCTATTCGGAAGGTACAGG	
F2B(*BamH* I)	CTAGGATCCAGCAGCATTATTACTATGTGC	
C-*stp17*-A(Xba I)	CGTCTAGAGGCAACTCCTTAGTTATG	pACYC184-*stp17* construction
C-*stp17*-B(*Hind* III)	GCAAGCTTGTAAGAGTCACCGGCATT	
O-*stp17*-A(*Nco* I)	GTACCATGGGTATGTTAGGGGGTTTTATGAT	pBAD-*stp17* construction
O-*stp17*-B(*Hind* III)	GCCAAGCTTTTATGCCTTCTCTTTTGCTTTC	
P-*stp17*-A(*Nco* I)	GACCATGGATATGTTAGGGGGTTTTATG	pET28b-*stp17* construction
P-*stp17*-B(*Xho* I)	ATCTCGAGTGCCTTCTCTTTTGCTTT	

Underline means the sequences recognized by specific restriction endonucleases.

### Construction of *stp17* mutant, complementation and over-expression strains

The *stp17* gene deletion mutant of *S.* Typhi was prepared through homologous recombination mediated by the suicide plasmid pGMB151, as previously described (Huang et al., 2004; Zhang et al., 2012a). The *stp17* deletion mutant was confirmed by sequencing analysis and designated as Δ*stp17*.

For complementary expression of *stp17* in Δ*stp17*, the CDS of *stp17* was amplified with *pfu* DNA polymerase by PCR. The amplicon was inserted into the complementary vector pACYC184 to form the recombinant plasmid pACYC184*-stp17*, which was verified by sequencing analysis. Then, the strain Δ*stp17* was transformed by the recombinant plasmid pACYC184*-stp17* and designated as the *stp17* complementary strain Δ*stp17*(pACYC184*-stp17*). As a control, the strain Δ*stp17* was also transformed with the empty vector pACYC184 and named Δ*stp17*(pACYC184).

The *stp17* ORF (a 642-bp DNA fragment) was cloned into the expression vector pBAD/gIII which can be induced by L-arabinose to generate the recombinant plasmid (pBAD-*stp17*). The recombinant plasmid pBAD-*stp17* was confirmed by sequence analysis and subsequently transformed into the wild type strain *S*. Typhi GIFU10007 to generate the over-expression strain wt(pBAD-*stp17*). As a control, the strain *S*. Typhi GIFU10007 was also transformed with the empty vector pBAD/gIII and named wt(pBAD). Over-expression of *stp17* in wt(pBAD-*stp17*) was induced by L-arabinose (0.1% wt/vol).

### Expression and purification of the recombinant protein STP17_*His*6_

The entire *stp17* ORF (a 642-bp DNA fragment) containing a *Nco* I site (5′-end) and a *Xho* I site (3′-end) was amplified by PCR. Then, the resulting PCR product was digested with *Nco* I and *Xho* I and cloned into plasmid pET28b which carries a N-terminal His-tag digested with the same enzymes. The resulting *stp17* recombinant expression plasmid pET28b-*stp17* was transformed into *E. coli* BL21(DE3) cells. The cell cultures were incubated at 37°C in LB medium until the OD_600_ reached 0.6. To induce expression of the recombinant protein, IPTG was added at a final concentration of 0.03 mM. The culture was grown for 5 h at 37°C and harvested by centrifugation (4000 r/min, 10 min, 4°C). Bacteria were re-suspended in 20 ml PBS. After bacteria were lysed with an ultrasonic cell disruptor, bacterial lysate was purified using a Ni-NTA agarose column as directed by the manufacturer (QIAGEN). Recombinant protein STP17_His6_ was eluted with elution buffer containing 250 mM imidazole. Purified STP17_His6_ from the elution buffer was concentrated in PBS using Amicon Ultra-15 Centrifugal Filter Unit with Ultracel-10 membrane, according to the manufacturer's protocol (Millipore Corporation, Bedford, MA, USA). Purified STP17_His6_ was analyzed by SDS-PAGE.

### Production of polyclonal antibody of STP17

To prepare the polyclonal antibody of STP17, purified STP17_His6_ was mixed completely with an equal volume of Freund's complete adjuvant and 1 ml of mixture which contained 50 μg STP17_His6_ was injected subcutaneously into each rabbit. After 1 week, purified STP17_His6_ was mixed completely with an equal volume of Freund's un-complete adjuvant and the mixture was used to immunize the rabbits as above every 2 weeks. The rabbits were immunized for total five times. Finally, the antiserum was obtained from the carotid artery, and was purified through salting out extraction with ammonium sulfate to prepare the rabbit polyclonal antibody of STP17.

### Verifying STP17 expression in *S*. Typhi GIFU10007 by western blot analysis

*S*. Typhi wild type and mutant Δ*stp17* strains were cultured overnight with shaking (250 rpm) at 37°C. Bacteria were normalized to OD_600_ 0.6. Proteins were separated by 15% SDS-PAGE and electrophoretically transferred to polyvinylidene difluoride (PVDF) membrane. The PVDF membrane was blocked with 5% dried skim milk. Rabbit anti-STP17 antiserum as the primary antibody was used at a dilution of 1:500 and incubated with the membrane for 2 h at room temperature. HRP-conjugated goat anti-rabbit-IgG antibody (Sigma-Aldrich, St. Louis, Missouri, USA) was used at a dilution of 1:10,000 as the secondary antibody. Horseradish peroxidase-antibody conjugates were detected by chemiluminesence.

### Investigation of STP17 expression in *S*. Typhi GIFU10007 by western blot analysis

The expression characteristics of STP17 protein in *S*. Typhi GIFU10007 under different growth phases were investigated by western blot. *S*. Typhi cells were collected when their OD_600_ values were 0.2, 0.5, 0.8, 1.2, and 1.6, respectively. Western blotting experiments in triplicate were performed as previously described.

### Plasmid curing and complementation of *stp17* into pBSSB1-dificient derivatives of *S*. Typhi

The plasmid curing method was referred to the reference (El-Mansi et al., 2000) with some following modifications. Sodium dodecyl sulfate (SDS) was added to LB media and the final concentration of SDS was adjusted to 1%. The wild-type strain *S*. Typhi GIFU10007 was inoculated into the above SDS containing LB and cultures were incubated overnight with shaking at 43°C. Subsequently, the cultures were inoculated into normal LB and incubated overnight with shaking at 43°C. The cultures were inoculated into SDS containing LB again and cultured overnight at 43°C with shaking. Then, the cultures were placed on to SS agar plates to select the possible pBSSB1-cured derivatives of *S*. Typhi through the amplification of the gene *fljB*^*z*66^ located on pBSSB1 by PCR. Finally, the possible pBSSB1-cured derivatives of *S*. Typhi were verified by Southern-blot as previous described (Zhang et al., 2012b) and designated as ΔpBSSB1. Then, the strain ΔpBSSB1 was transformed by the recombinant plasmid pACYC184*-stp17* and designated as the *stp17* complementary ΔpBSSB1 strain ΔpBSSB1(pACYC184*-stp17*). As a control, the strain ΔpBSSB1 was also transformed with the empty vector pACYC184 and named ΔpBSSB1 (pACYC184).

### Structure prediction of STP17 and mutagenesis of *stp17* gene

Based on the amino acid sequence of protein STP17 which were retrieved from GenBank (accession no. AM419040.1; Baker et al., 2007a), its secondary structure and three-dimensional structure were predicted by I-TASSER online server (http://zhanglab.ccmb.med.umich.edu/I-TASSER/) as previous described (Cai et al., 2013). In addition, the conserved ATPase domain of STP17 was predicted, and the possible ATP binding site residues of STP17 was analyzed through I-TASSER online server as well. According to the predicted possible ATP binding site residues of STP17, the site-directed mutations were introduced into pACYC184-*stp17* using standard PCR mutagenesis techniques, and mutations were confirmed by DNA sequencing. The mutants generated were the following: Δ*stp17*:: pACYC184-*stp17*^G16A^, Δ*stp17*:: pACYC184-*stp17*^G17A^, Δ*stp17*:: pACYC184-*stp17*^G19A^, Δ*stp17*:: pACYC184-*stp17*^K20A^, Δ*stp17*:: pACYC184-*stp17*^N133A^, Δ*stp17*:: pACYC184-*stp17*^R134A^, Δ*stp17*:: pACYC184-*stp17*^K157A^, Δ*stp17*:: pACYC184-*stp17*^K158A^.

### Growth curve assay

Bacteria were grown in LB medium at 37°C. A single colony of bacteria from a LB agar plate was inoculated into 2 ml of LB broth and incubated at 37°C with shaking (250 rpm) overnight. Then, the culture was diluted 1/100 in fresh LB broth (containing L-arabinose (0.1% wt/vol) for the culturing of strains harboring pBAD). Cell growth was monitored at OD_600_ every hour using a BioPhotometer (Eppendorf, Hamburg, Germany). The experiments were repeated at least three times.

### Motility assay

For detecting the motility of bacteria, different strains were incubated until their OD_600_ reached 0.4. From each culture, 2 μl was inoculated onto a 0.3% semisolid LB agar plate (containing L-arabinose 0.1% wt/vol) for the strains harboring pBAD). The plates were incubated at 37°C for 10 h and motility was assessed qualitatively by examining the diameter of circular swimming, which was formed by growing motile bacterial cells. Meanwhile, the flagella gene *fljB*^*z*66^ and *fliC* expression of different strains were examined by qRT-PCR.

### STP17 ATPase activity assay

The potential ATPase activity of STP17 was determined by the ATP assay kit (Beyotime, China) according to the manufacturer's recommended protocol. Briefly, STP17_His6_ protein was added to the ATP solution provided in the assay kit at a concentration of 10 μM and then incubated at 25°C for 10-, 30-, and 50 min, respectively. The ATP concentration of the above reaction mixtures was measured by a F-4500 fluorescent spectrophotometer (Hitachi, Japan). As a control, BSA protein which lacks ATPase activity was mixed with the ATP solution and analyzed on the spectrophotometer. Because ATPase can catalyze ATP into ADP and free phosphate, the reduction of ATP concentration in the reaction mixture indicates the presence of ATPase activity, which should be dependent on the added protein being studied (STP17_His6_). These experiments were performed at least three times.

### Statistical analysis

Data were analyzed using Prism 5 software. The Student's *t*-test was used to determine significant differences between results. Significance was defined as *P* < 0.05.

## Results

### The gene *stp17* is expressed and translated into STP17 protein in *S.* Typhi

**Figure 1:**
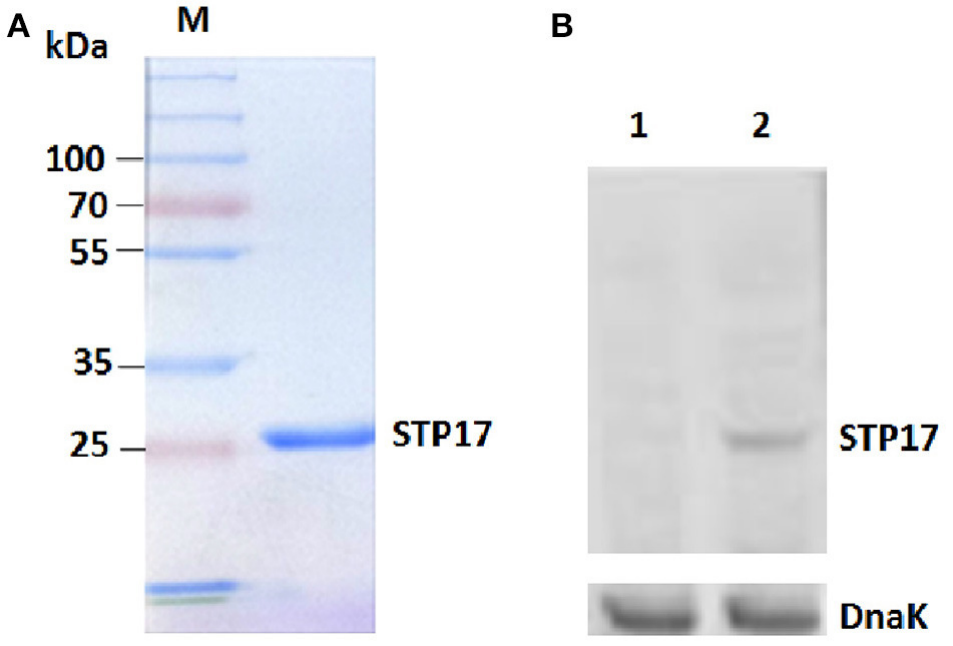
**Identification of *stp17* expression in *S.* Typhi**. **(A)** SDS-PAGE analysis of STP17_His6_ protein purification; **(B)** Western blot analysis using the purified anti-STP17_His6_ polyclonal antibody. Lane 1, Δ*stp17*; Lane 2, *S*. Typhi GIFU 10007. DnaK: the loading control marker.

The gene *stp17* is located on a linear plasmid named pBSSB1 in *S*. Typhi and is predicted to encode a putative nucleotide binding protein, STP17. In this study, expression of *stp17* in *E.* coli BL21(DE3) was generated with the expression vector pET28b. Results showed that the purified recombinant protein STP17_His6_ was successfully obtained (Figure 1A). To verify whether the *stp17* gene is expressed and translated into STP17 protein in *S.* Typhi, western blotting was performed to identify STP17 levels in the *S*. Typhi wild type strain and the mutant strain Δ*stp17* (Figure 1B). The results showed that the gene *stp17* can be translated into STP17 protein in *S.* Typhi.

### Protein expression characteristics of STP17 in different growth phases

**Figure 2:**
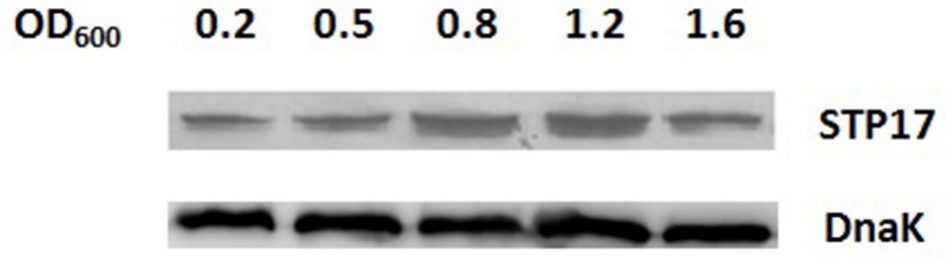
**Western blot analysis of STP17 protein levels in *S*. Typhi in different growth phases**. OD_600_ values (0.2, 0.5, 0.8, 1.2, and 1.6) corresponding to early log phase, mid-log phase, late log phase, early stationary phase and late stationary phase, respectively. DnaK: the loading control marker.

We investigated the expression characteristics of STP17 in different growth phases by western blot analysis. Five OD_600_ values (0.2, 0.5, 0.8, 1.2, and 1.6) corresponding to early log phase, mid-log phase, late log phase, early stationary phase and late stationary phase, respectively, were monitored. As shown in Figure 2, *stp17* can be expressed stably in normal growth conditions with expression levels increasing with OD_600_ values throughout log phase. The expression level of STP17 reaches a peak value at an OD_600_ value of 1.2. Then, expression levels decrease with OD_600_ values at stationary phase.

### The gene *stp17* affects the growth of *S*. Typhi in a pBSSB1 independent manner

**Figure 3:**
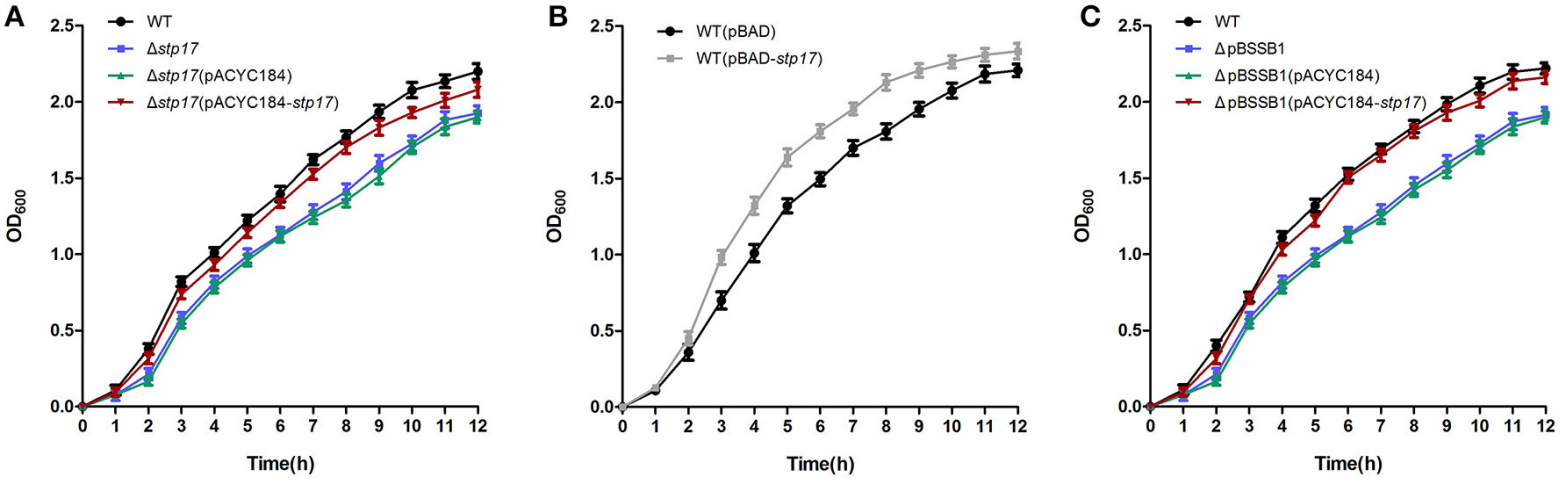
**Effect of *stp17* on the growth rate of *S.* Typhi. (A)** Growth curve of wild type strain, *stp17* deletion mutant and *stp17* complementary strain. **(B)** growth curve of over-expression strain. **(C)** growth curve of wild type strain, pBSSB1-cured derivative and pBSSB1 complementary strain.

To investigate the role of the *stp17* gene in *S*. Typhi, the *stp17* mutant was constructed by homologous recombination mediated by the suicide plasmid. We measured growth of the wild-type and Δ*stp17* strain and found that growth of *stp17* mutant strain was significantly slower compared to wild type (*P* < 0.05; Figure 3A). In addition, the growth of complementary strain Δ*stp17*(pACYC184*-stp17*) restored to the wild type level (Figure 3A). At the same time, we constructed the *S*. Typhi *stp17* over-expression strain wt(pBAD-*stp17*) and found that cell growth upon STP17 over-expression was significantly increased compared with the wild type strain (*P* < 0.05; Figure 3B). As shown in Figure 3C, the growth of pBSSB1-deficient strain ΔpBSSB1 was obviously decreased compared with the wild type strain because *stp17* was absent due to the loss of pBSSB1. However, the growth rate of *stp17* complementary ΔpBSSB1 strain ΔpBSSB1(pACYC184*-stp17*) was similar to the wild type strain. In addition, we compared the stability of pBSSB1 in wild type and Δ*stp17*, and found no obvious difference presenting after deletion of *stp17* (data not shown). These results suggest that *stp17* has an effect on *S.* Typhi cell growth in a pBSSB1 independent manner.

### The gene *stp17* enhances the motility of *S.* Typhi in a pBSSB1 independent manner

**Figure 4:**
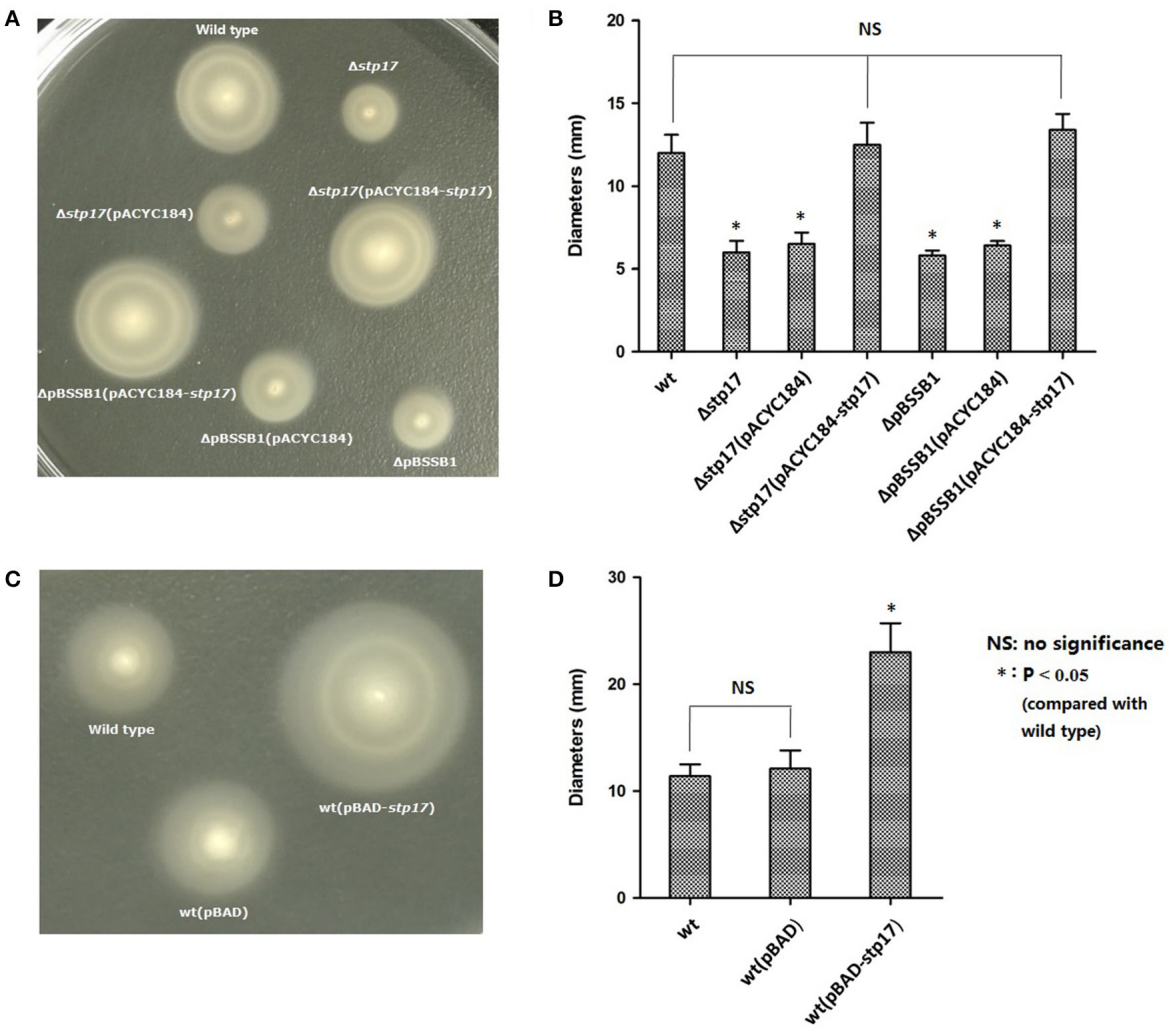
**Motility ring and diameter of *S*. Typhi. (A,B)** Effect of *stp17* and pBSSB1 on the motility of *S*. Typhi. **(C,D)** Effect of *stp17* over-expression on the motility of *S*. Typhi.

As shown in Figure 4A, the motility of Δ*stp17* was greatly decreased compared with the wild type strain, and the motility of pBSSB1-deficient strain ΔpBSSB1 was obviously decreased as well because *stp17* was absent due to the loss of pBSSB1. However, bacterial motility was restored obviously in the complementary strain Δ*stp17*(pACYC184*-stp17*) and ΔpBSSB1(pACYC184*-stp17*). Moreover, the motility of *stp17* over-expression strain wt(pBAD-*stp17*) was much greater compared with the control strain wt(pBAD) (Figure 4C). The ring diameters for various strains are shown in Figures 4B,D. Moreover, the expression of flagellar gene *fljB*^*z*66^ and *fljC* show no obvious difference when *stp17* was deleted (data not shown). This result suggests that the differences of bacterial motility may be not due to the different expression of flagellar gene. The data demonstrate that *stp17* affects the motility of *S.* Typhi in a pBSSB1 independent manner.

### Structure prediction of STP17

**Figure 5:**
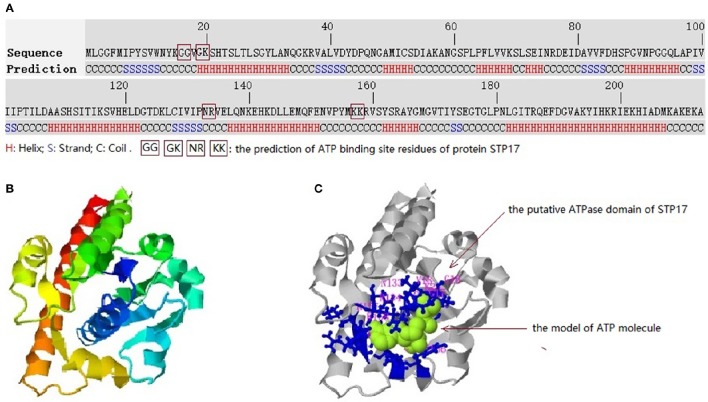
**Predicted 3-D structures and conserved domains of the protein STP17. (A)** The amino acid sequence of STP17 and the predicted secondary structure. **(B)** The top-ranked 3-D structure of STP17 predicted by I-TASSER. **(C)** The putative ATPase domain, the ligand molecule of STP17 is indicated by green and the top-ranked predicted active residues GLY16, GLY17, GLY19, LYS20, ASN133, ARG134, LYS157, and LYS158 is shown by blue.

The structure of STP17 was predicted by I-TASSER online server, as explained in Materials and Methods. As shown in Figure 5A, the 213 amino acids of STP17 contains nine Helix segments, six Stand segments and some Coil segments in the secondary structural level. The top-ranked 3-D structure of STP17 predicted by I-TASSER was shown in Figure 5B, and it contains a putative ATPase domain like a pocket (Figure 5C), with the top-ranked predicted residues GLY16, GLY17, GLY19, LYS20, ASN133, ARG134, LYS157, and LYS158 as ATPase active site residues (Figure 5A).

### Identification of the active site residues of STP17

**Figure 6:**
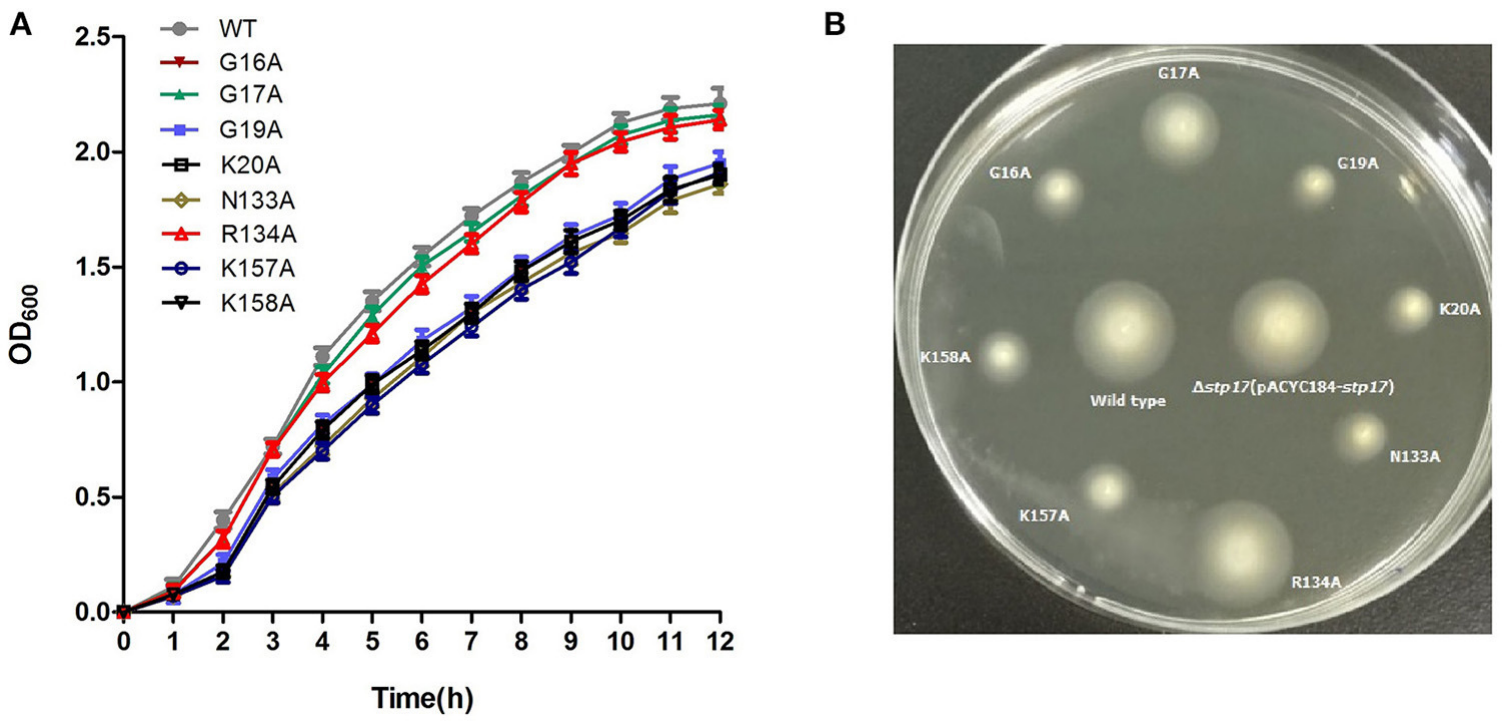
**Identification of the active site residues of STP17**. **(A)** Growth cure of site-directed mutants of STP17. **(B)** Motility comparison of site-directed mutants of STP17. G16A, G17A, G19A, K20A, N133A, R134A, K157A, and K158A represent eight mutants through site-directed mutagenesis.

To define the active site of STP17 activity, eight mutants of site-directed mutagenesis were generated, and their growth and motility were compared to the wild type strain. As shown in Figure 6, when the residues of GLY16, GLY17, GLY19, LYS20, ASN133, ARG134, LYS157, and LYS158 mentioned above were mutated, the growth and motility of these mutant strains Δ*stp17*::pACYC184-*stp17*^G16A^, Δ*stp17*::pACYC184-*stp17*^G19A^,_Δ*stp17*::pACYC184- *stp17*^K20A^,_ Δ*stp17*::pACYC184- *stp17*^N133A^,_Δ*stp17*::pACYC184- *stp17*^K157A^, and Δ*stp17*::pACYC184-*stp17*^K158A^ were significantly decreased compared with that of wild type strain, while there was no obviously change in growth and motility of mutant strains Δ*stp17*::pACYC184-*stp17*^G17A^ and Δ*stp17*::pACYC184-*stp17*^R134A^. In addition, the stability of wild type strain and above mutants shows no significant difference (data not shown). All these results suggest that the amino acid residues of GLY16, GLY19, LYS20, ASN133, LYS157, and LYS158 may be the active site residues of STP17.

### Analysis of ATPase activity of STP17

**Figure 7:**
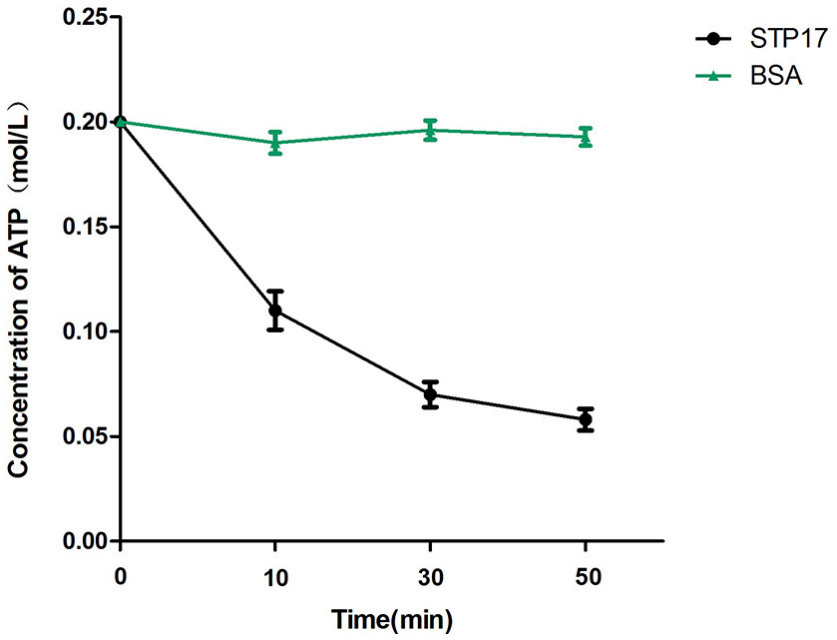
**Analysis of ATP reduction by STP17**. BSA (Bovine Serum Albumin), which lacks the ATPase activity, was used as a negative control.

To identify the potential ATPase activity of STP17, we investigated whether the ATP concentration in solution could be decreased in the presence of STP17. As shown in Figure 7, the concentration of ATP in solution decreased markedly after incubation with STP17 for 10 min and continued to decrease after incubation for 30- and 50-min, while the concentration of ATP in solution containing the control protein BSA which lacks the ATPase activity did not decreased. The reduction in ATP concentration indicates catalytic activity of STP17, suggesting that STP17 can catalyze ATP into ADP and free phosphate.

## Discussion

Plasmids are extra chromosomal, self-replicating genetic elements with additional functions, such as antibiotic resistance, that are complementary to chromosomal DNA. In many cases, bacterial plasmid DNA is circular. In 1979, the first linear plasmid in a prokaryote was found in *Streptomyces rochei* (Hayakawa et al., 1979). Subsequently, another kind of linear plasmid was also found in *Borrelia* (Plasterk et al., 1985). However, most linear plasmids have been found in about a dozen of *Streptomyces*, and their molecular size is between 12 and 640 kb (Hinnebusch and Tilly, 1993; Zhong et al., 2010). In 2007, Baker et al. reported an approximately 27 kb linear plasmid named pBSSB1 in the *S.* Typhi z66-positive strain and indicated this plasmid mediates unidirectional flagellar phase variation (Baker et al., 2007a,b). The pBSSB1 plasmid is the first non-bacteriophage-related linear plasmid found in *Enterobacteriaceae* (Baker et al., 2007a). It was found that the factor for inversion stimulation (Fis) encoded by the *fis* gene can affect the stability of pBSSB1 in *S.* Typhi (Zhang et al., 2012b). There are 33 putative ORFs on pBSSB1 including the operon *fljBA*^*z*66^, which encodes the phase-2 flagellin FljB^*Z*66^ and the repressor FljA^*Z*66^ of the phase-1 flagellin gene *fliC*, respectively (Huang et al., 2004; Baker et al., 2007a,b; Zou et al., 2009). There is also a notion that most of these genes encoded on pBSSB1, except the *fljBA*^*z*66^, are pseudogenes (Simon et al., 1980).

In this study, the gene *stp17* was cloned and expressed successfully in *E. coli*. The purified STP17_His6_ protein was gained and the polyclonal anti-STP17 antibody was prepared in rabbit. Then, the anti-STP17 antibody was used to investigate whether the *stp17* gene is expressed at the protein level through western blot analysis. The results showed that STP17 was expressed well in the wild type strain of *S*. Typhi. Gene expression of *stp17* has already been verified by qRT-PCR and Northern-blot (Zhao et al., 2014). Therefore, the gene *stp17* is not a pseudogene and can be expressed at both the mRNA and protein levels.

In 2014, we investigated the transcriptional expression of several genes located on pBSSB1 in different growth phases and environmental stresses and found that the expression of *stp17* increases with the OD_600_ value in log phase and is not influenced by acidic stress, oxidative stress or osmotic stress (Zhao et al., 2014). Here, the translational expression level of *stp17* was studied by western blot analysis. It was found that the expression level of STP17 increased with the OD_600_ value throughout log phase and reached a peak value at an OD_600_ of 1.2, then decreased in stationary phase. The expression profile of *stp17* at the mRNA and protein level under different growth phases is very similar and shows that *S*. Typhi needs more STP17 when cells are in the growth period. When *stp17* was deleted, growth of the Δ*stp17* strain was obviously slower than the wild type strain. Moreover, cell growth under *stp17* over-expression was faster than the control strain. Therefore, STP17 may be involved in promoting bacterial growth.

The DNA sequence of pBSSB1 shows that the *stp17* gene is supposed to encode a putative nucleotide binding protein and it is immediately adjacent to the possible replication origin of pBSSB1 (Baker et al., 2007a). In addition, *stp17* is predicted to encode a protein containing a conserved domain, which is shared by the ParA protein. ParA is involved in the segregation of plasmids and bacterial chromosomal DNA (Motallebi-Veshareh et al., 1990; Volante and Alonso, 2015). Therefore, *stp17* is suggested to be involved in the replication of pBSSB1. Although, it was shown that the stability is not affected by *stp17* in this study, future studies are required to determine whether *stp17* is involved in replication or segregation of pBSSB1.

The amino acid sequence of STP17 was analyzed in this study and an ATPase domain was predicted. The predicted activity of STP17 was also identified in this study. Moreover, ParA, which shares a conserved domain with STP17, was reported to possess the sequence of a conserved and widespread family of ATPases (Motallebi-Veshareh et al., 1990; Bignell and Thomas, 2001). ATPases is very important to bacterial motility (Bai et al., 2014; Lin et al., 2015). In bacteria, a specific protein export apparatus, which utilizes ATP and proton motive force as the energy source to transport component proteins to the distal growing end, is crucial for self-assembly of the bacterial flagellum (Erhardt et al., 2014; Minamino et al., 2014). It was reported that bacterial motility was obviously reduced due to infrequent ATP hydrolysis caused by mutation of the FliI6-FliJ complex, which is similar to the FOF1-ATPase (Imada et al., 2016; Minamino et al., 2016). It was shown that the motility of Δ*stp17* was significantly decreased compared to the wild type strain in this study. One explanation for this phenotype is that STP17 could utilize ATP and proton motive force as the energy source through its ATPase activity to help bacterial motility.

In summary, this study is the first to show that the *stp17* gene is not a pseudogene and is expressed well at the protein level. Furthermore, our results show that *stp17* plays an important role in promoting the growth and motility of bacteria.

## Author contributions

Conceived and designed the experiments: HZ, XH, HX. Performed the experiments: HZ, YuZ, XX, MW, YiZ, MG, BN. Analyzed the data: HZ, YuZ, HD, SX. Wrote the manuscript: HZ, YuZ, XH.

### Conflict of interest statement

The authors declare that the research was conducted in the absence of any commercial or financial relationships that could be construed as a potential conflict of interest.
